# Pharmacokinetic Behavior of Vincristine and Safety Following Intravenous Administration of Vincristine Sulfate Liposome Injection in Chinese Patients With Malignant Lymphoma

**DOI:** 10.3389/fphar.2018.00991

**Published:** 2018-08-29

**Authors:** Fen Yang, Min Jiang, Ming Lu, Pei Hu, Hongyun Wang, Ji Jiang

**Affiliations:** ^1^Key Laboratory of Carcinogenesis and Translational Research (Ministry of Education), Center of Drug Clinical Trial, Peking University Cancer Hospital and Institute, Beijing, China; ^2^Clinical Pharmacology Research Center, Peking Union Medical College Hospital, Chinese Academy of Medical Sciences, Beijing, China; ^3^Beijing Key Laboratory of Clinical PK and PD Investigation for Innovative Drugs, Beijing, China

**Keywords:** free vincristine, total vincristine, pharmacokinetics, vincristine sulfate liposome injection, urinary excretion, safety

## Abstract

**Objective:** This phase Ia study was designed to assess the pharmacokinetic (PK) characters of free vincristine (F-VCR, refer to as non-liposomal VCR and VCR released from liposome) and total vincristine (T-VCR, the sum of both liposomal VCR and F-VCR), urinary excretion and safety of intravenous administration of vincristine sulfate liposomes injection (VSLI) in Chinese patients with malignant lymphoma and compare the results with those for conventional vincristine sulfate injection (VSI).

**Methods:** In the phase Ia, randomized, open-label, two sequence cross-over study, patients from one group were exposed to treatment 1 including cytoxan (cyclophosphamide power injection), hydroxyrubicin (adriamycin power injection), oncovin (VSI), and prednisone tablets (standard CHOP scheme) before crossed over to treatment 2 (modified CHOP scheme in which VSI was replaced with VSLI). Patients from another group received treatments in reverse order.

**Results:** In this phase Ia study, a total of eight subjects participated. VCR elimination from the circulation after injection of VSLI was characterized by a significantly increased maximum concentration (C_max_, 86.6 ng/mL) and plasma area under the plasma concentration-time curve from zero to infinity (AUC_0-Inf_, 222.1 ng/mL h), markedly decreased distribution volume (V_z_, 224.1 L) and plasma clearance (CL, 8.9 L/h) compared to lower C_max_ (26.6 ng/mL) and AUC_0-Inf_ (95.1 ng/mL h), larger V_z_ (688.8 L) and CL (22.1 L/h) for VSI. The small proportion of F-VCR following infusion of VSLI in circulation was reflected by very low C_max_ (1.8 ng/mL) and AUC_0-Inf_ (50.5 ng/mL h). Less than 3% of the administered dose of VSLI was excreted in urine and the extent was similar to that for VSI. The elimination percentage of 40–21–14% for VSI changed to 6.2–24–39% for VSLI at intervals of 0–5, 5–13 and 13–25 h, respectively. Significant difference of toxicity between VSLI and VSI was not observed.

**Conclusion:** VSLI exhibits higher AUC_0-Inf_ of T-VCR, lower CL and V_z_ compared with VSI. VSLI was well tolerated, maybe due to the markedly decreasing AUC_0-Inf_ of F-VCR. The majority of VCR was enveloped in liposome and VCR was released gradually from liposome following injection of VSLI. Liposomal encapsulation of VCR does not alter the route and extent of VCR excretion in urine.

## Introduction

Vincristine sulfate (VCR) remains a potent and widely used antitumor agent for more than 50 years (Johnson et al., 1963) and it has significant activity against a non-Hodgkin lymphoma (NHL) subtypes and acute lymphoblastic leukemia (ALL) (Gidding et al., 1999). VCR exhibits cell cycle-specific cytotoxic activity by binding to tubulin, resulting in microtubule depolymerization, metaphase arrest and apoptosis in cells (Schou et al., 1968; Owellen et al., 1972, 1976). Thus, the antitumor efficiency of VCR is dependent on the drug concentration and duration of exposure at the tumor site (Horton et al., 1988). However, the routinely individual standard VCR dose is limited to 1.4 mg/m^2^ or a maximum 2 mg (i.e., dose capping) in most VCR-containing cancer treatment regimens in order to reduce the risk of severe peripheral and central nervous system (CNS) neurotoxicity (Hildebrand et al., 1972; Legha, 1986), which may limit its optimal clinic benefit.

Liposomes are versatile drug carriers that allow effective delivery of drug to target tissue, prolong the circulation time of encapsulated drug and slowly release the drug, resulting in high levels of encapsulated drug in target tissues and a long duration of exposure of tumor cells to therapeutic drug concentrations (Drummond et al., 1999; Allen et al., 2006). Vincristine sulfate liposome injection (VSLI) is an encapsulated preparation of standard VCR in liposomes, which was designed to overcome the dosing and PK limitations of conventional vincristine sulfate injection (VSI). VSLI has been studied extensively both in laboratory (Kanter et al., 1994; Webb et al., 1995, 1998; Krishna et al., 2001; Zhong et al., 2014; Shah et al., 2016a) and in the clinic (Embree et al., 1998; Gelmon et al., 1999; Bedikian et al., 2006, 2011; Rodriguez et al., 2009; Thomas et al., 2009; Yan et al., 2012; Silverman et al., 2013; Douer, 2016; Shah et al., 2016b) to show the superiority over standard VCR. Marqibo^®^ (Hana Biosciences, Inc.) was the first listed VSLI approved by FDA at 2012 (Silverman and Deitcher, 2013). Although higher antitumor activity with good tolerance was observed for VSLI, a clear understanding of concentration-effect/toxicity relationship, the release properties of VCR from liposome and the contribution of F-VCR to the overall PK profile of T-VCR for VSLI in human being are still limited.

In the present study, a new VSLI (developed by Shanghai Fudan-zhangjiang Bio-Pharmaceutical Co.) was introduced in the phase Ia trial, which was conducted to study the PK characters of F-VCR and T-VCR, the VCR urinary excretion and the safety in Chinese subjects with malignant lymphoma after VSLI injection.

## Materials and Methods

### Materials

Vincristine sulfate liposome injection (0.16 mg/mL) was provided by Shanghai Fudan-zhangjiang Bio-Pharmaceutical Co., Ltd., China, which is a three-part formulation containing empty liposomes, disodium hydrogen phosphate and VCR sulfate for injection. After the standard procedure, the encapsulation efficiency of liposomal VCR was up to 95% and liposomal VCR remains stable for at least 24 h when stored at 4°C. VSI, cytoxan (cyclophosphamide power injection), hydroxyrubicin (adriamycin power injection), oncovin (VSI) and prednisone tablets were purchased through Nanjing Pharmaceutical Hefei Tianxing Co., Ltd., China.

### Study Subjects

Eligible patients aged from 18 to 65 years had histologically or cytologically confirmed malignant lymphoma, which were refractory to conventional forms of cancer therapy. They should not receive other therapy including surgery, chemotherapy or radiotherapy within at least previous 4 weeks. In addition, it was required for eligible patients to have life expectancy of at least 12 weeks, and Eastern Cooperative Oncology Group performance status of 0 to 2, and adequate bone marrow function (leukocyte count ≥4.0 × 10^9^/L, an absolute granulocyte count ≥1.5 × 10^9^/L, a platelet count 75.0 × 10^9^/L and a hemoglobin count ≥80g/L), and adequate hepatic function (alanine aminotransferase [ALT], aspartate aminotransferase [AST] and total bilirubin ≤3 × the upper limit of normal [ULN], and total bilirubin ≤1.5 × ULN), and renal function (serum creatinine ≤1.5 × ULN).

Patients with brain metastases, or CNS disorder, or severe cardiac and cerebral vascular diseases, or active infection were excluded. Patients who were allergic to the test drug and accessories were ineligible.

### Study Design

**FIGURE 1:**
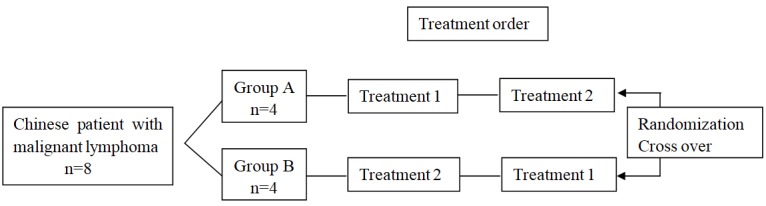
Study design. Treatment 1, standard CHOP scheme including cytoxan (cyclophosphamide power injection, 750 mg/m^2^), hydroxyrubicin (adriamycin power injection, 50 mg/m^2^), oncovin (VSI, 1.4 mg/m^2^) and prednisone tablets (100 mg); Treatment 2, modified CHOP scheme in which VSLI (1.0mg/m^2^) replaced VSI. The drugs for injection were administrated intravenously (I.V.) on study day 1 and the tablets were swallowed whole for concessive 5 days from day 1 to 5 of each 21-day circle.

The study design was shown in **Figure 1**. In the Phase Ia study with multi-center, randomized, open-label and cross-over design, two treatments were administered under fasted conditions. The treatment sequence for each participant was assigned by a computer-generated randomization list. Treatment 1 was standard CHOP scheme including cytoxan (cyclophosphamide power injection, 750 mg/m^2^), hydroxyrubicin (adriamycin power injection, 50 mg/m^2^), oncovin (VSI, 1.4 mg/m^2^) and prednisone tablets (100 mg). The drugs for injection were administrated intravenously (I.V.) on study day 1 and the tablets were swallowed whole for concessive 5 days from day 1 to 5 of each 21-day circle. Treatment 2 was modified CHOP scheme in which VSLI (1.0 mg/m^2^) replaced VSI and others remained unchanged. VSI and VSLI should be administrated within 60 ± 5 min.

Subjects fasted overnight for at least 10 h prior to drug administration and for at least 2 h post-dose, after which controlled food intake was allowed. Cigarette, alcohol, tea, coffee, grapefruit-, or drug-containing food or drink, and non-investigator-approved prescription medications or over-the-counter products were to be avoided at least 24 h post-dose and during the study.

### Blood and Urine Sampling for PK Analyses

Blood samples were collected at pre-dosing, 15, 30, and 60 min after infusion and 0.25, 0.5, 2, 4, 8, 12, 24, 48, 72, and 96 h after the end of infusion. The whole blood was centrifuged (450 ×*g* at 4°C for 10 min) and obtained plasma then was divided into three parts. One part was used to separate F-VCR from liposomes using solid phase extraction (SPE) method immediately and the remaining two parts (one part was used as the backup) were frozen for assay of the T-VCR concentration.

Urine samples were collected from four patients of group A (one female and three males) before and after drug infusion up to 97 h at six intervals: 0–5, 5–13, 13–25, 25–49, 49–73, and 73–97 h. The samples from each interval were mixed and the volume was measured. Then 10 mL urine samples were remained and frozen at -80°C.

### Bioanalytical Methods

The plasma concentration of F-VCR and T-VCR and the urine VCR concentration was determined using the ultra-performance liquid chromatography-tandem mass spectrometry (UPLC-MS/MS) system (Waters Corporation, Milford, MA, United States) with vinblastine sulfate used as the internal standard (IS) as previously described by our team (Yang et al., 2013, 2015). The compounds were detected as doubly charged ions and the multiple reaction monitor (MRM) transitions of VCR and the IS were *m/z* 413.2 → 353.2 and *m/z* 406.2 → 271.6, respectively. F-VCR was separated from liposomal form in plasma using SPE method; plasma T-VCR and urine VCR were extracted using liquid-liquid extraction method. F-VCR and T-VCR were identified and quantified over a theoretical concentration range of 0.2–50 ng/ml for F-VCR in plasma, 0.5–400 ng/ml for T-VCR in plasma and 0.5–100 ng/ml for VCR in urine, respectively. Assay specificity was assessed using blank sample from six different lots to verify the absence of interference at retention time. Quantitation was made using peak area ratios of analyte/IS, and back-calculated concentrations were determined using a weighted (1/ × 2) linear regression (y = ax + b).

### Data and Statistical Analyses

The measured plasma concentration was used to obtain C_max_ and the time to reach C_max_ (T_max_) directly. The main PK parameters, calculated from the plasma concentration-time data using a non-compartmental analysis method (WinNonlin Professional Network Edition, Version 7.0, Pharsight Corp., Palo Alto, CA, United States), were elimination half-life (t_1/2_), the area under plasma concentration-time curve from zero to last time (AUC_0-t_) and from zero to infinity (AUC_0-Inf_), the mean retain time from zero to last time (MRT_0-t_) and from zero to infinity (MRT_0-Inf_), clearance (CL) and apparent volume of distribution (V_d_). Mann-Whitney test was used to compare PK parameters (C_max_, AUC_0-t_, t_1/2_, V_z_ and CL) between the two groups using GraphPad Prism software (Version 7.0). *P*-value less than 0.05 was considered to be significant.

### Safety Assessments

The safety of the subjects was monitored by evaluation of physical examinations, vital signs, electrocardiograms, and clinical laboratory tests and adverse events (AEs) reporting. AEs were classified by system organ class and were graded according to National Cancer Institute (NCI) Common Terminology Criteria for Adverse Events (CTCAE), version 4. Safety results were analyzed using descriptive statistics.

## Results

### Subjects

There were eight patients (four males and four females) enrolled in this study. Other demographic data are shown in **Table 1**. Nobody withdrew/were withdrawn before the end of the study. All patients were assessable for safety. All subjects provided measurable PK data and were therefore included in the PK evaluation and statistical analysis. **Table 2** is a summary of dose level, number of the patients and actual dose delivered.

**Table 1:** Demographic characteristics of the patients.

		Group A (*n* = 4)	Group B (*n* = 4)
Sex			
	Male	3	1
	Female	1	3
Age (years)		54.9 (45.6–62.0)	48.2 (20.0–70.3)
Body surface area (m^2^)		1.7 (1.3–1.8)	1.8 (1.5–1.9)
ECOG status			
	0–1	4	4
	2	0	0
Tumor types			
	Malignant lymphoma	4	4
	Prior therapy		
	Surgery	2	0
	Chemotherapy	4	4

**Table 2:** Planned dose levels and doses delivered.

Dose level (mg/m^2^)	Number of patients enrolled	Vincristine dose delivered (mg)	
		Median	Range
1.4 VSI	8	2.00	2.00–2.00
1.0 VSLI	8	1.75	1.33–1.89

### PKs of T-VCR

**FIGURE 2:**
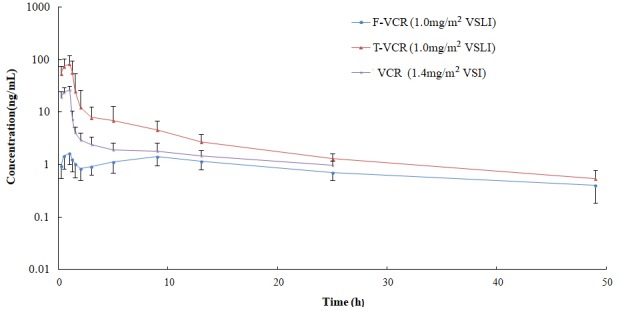
Mean logarithmic concentration vs. time plot of T-VCR and F-VCR following infusion of VSLI (1.0 mg/m^2^) and VCR post injection of VSI (1.4 mg/m^2^).

The mean plasma T-VCR concentration vs. time curves of patients who received VSLI (1.0 mg/m^2^) or VSI (1.4 mg/m^2^) are shown in **Figure 2**. The corresponding PKs of T-VCR were calculated from these data and are presented in **Table 3**. The maximum plasma T-VCR concentrations (C_max_) were observed at the end of the VSLI infusion, subsequently a rapid distribution phase occurred, in which concentration rapidly declined within 1 h, followed by a slow distribution phase and a terminal elimination phase. Therefore, the T-VCR plasma concentrations for all subjects were characterized by a triexponential decline after treated with VSLI. Although the profiles for VSI are very similar to those for VSLI, VSI exhibited significant lower C_max_ and AUC_0-Inf_ of VCR, as well as markedly higher V_z_ and CL than those of T-VCR for VSLI. After administration of VSLI, significantly elevated systematic exposure (C_max_ was 86.6 ng/mL and AUC_0-Inf_ was 222.1 ng/mL h), associated with decreased CL (8.9 L/h) and V_z_ (224.2 L) of T-VCR were obtained, compared with those of VCR for VSI (*P* < 0.01). There was no significant difference for half life (t_1/2_) between two groups (VSLI, 18.4 h vs. VSI, 22.5 h, *P* = 0.065).

### PKs of F-VCR

The mean plasma F-VCR concentration-time plots of patients after administration with VSLI at dose level of 1.0 mg/m^2^ is shown in **Figure 2**. Key PK parameters of plasma F-VCR are shown in **Table 3**.

**Table 3:** Main PK parameters of F-VCR and T-VCR following infusion of VSLI (1.0 mg/m^2^) and VCR post injection of VSI (1.4 mg/m^2^).

Parameters	VSLI (1.0 mg/m^2^)		VSI (1.4 mg/m^2^)
	F-VCR (*n* = 8)	T-VCR (*n* = 8)	VCR (*n* = 8)
C_max_ (ng/mL)	1.8 (0.6)	86.6 (37.4)^a^	26.6 (5.2)
AUC_0-t_ (ng/m⋅h)	50.5 (21.8)	207.6 (86.3)^b^	74.4 (20.3)
AUC_0-Inf_ (ng/mL⋅h)	64.8 (32.9)	222.1 (86.5)^c^	95.1 (22.1)
T_max_ (h)	1.9 (3.0)	0.9 (0.3)	0.9 (0.2)
t_1/2_ (h)	35.6 (16.2)	18.4 (10.2)^d^	22.5 (5.7)
MRT_0-t_ (h)	28.7 (9.1)	8.0 (4.1)	10.6 (3.8)
MRT_0-Inf_ (h)	50.0 (21.1)	14.1 (8.8)	24.1 (6.8)
CL (L/h)	30.9 (11.8)	8.9 (4.2)^e^	22.1 (6.5)
Vz (L)	1364.9 (398)	224.1 (109)^f^	688.8 (131.2)

*Values are expressed as mean (SD). ^a^P = 0.0002 vs. VSI group. ^b^P = 0.0006 vs. VSI group. ^c^P = 0.0011 vs. VSI group. ^d^P = 0.065 vs. VSI group. ^e^P = 0.0006 vs. VSI group. ^f^P = 0.0002 vs. VSI group.*

Multiple peaks were apparently observed for the individual F-VCR concentration-time curve (**Supplementary Material**). The peak concentration (C_max_) of F-VCR was obtained at the end of VSLI infusion (1 h) in most patients (7 of 8 patients). Another one or two lower concentration peaks appeared at 9 or 3/9 h after the start of infusion. Then, the F-VCR concentration gradually declined. At 1 h after i.v. administration of VSLI, the mean plasma concentration of F-VCR was 1.8 ng/ml, compared with 86.6 ng/ml for T-VCR. This indicated that F-VCR represented 2% of the T-VCR in the plasma at this time point. The proportion of F-VCR in the plasma was evaluated by comparing the AUC values. The AUC_0-Inf_ value for F-VCR after the administration of VSLI was 64.8 ng/mL h, which represent 29.2% of the AUC_0-Inf_ for T-VCR. In addition, systemic exposure of F-VCR for VSLI was compared with that of VCR for VSI. Although the similar AUC_0-Inf_ value of F-VCR to that of VCR was obtained after dose calibration, the lower F-VCR concentration at every time point from 0.25 to 25 h after administration of VSLI than that of VCR for VSI was observed. It is important to note that the significant decreased C_max_ of F-VCR contrasts the increased plasma VCR concentration observed after VSI administration where peak value was 26.3 ng/mL, 16-fold greater than that of F-VCR.

### Urinary Excretion

**FIGURE 3:**
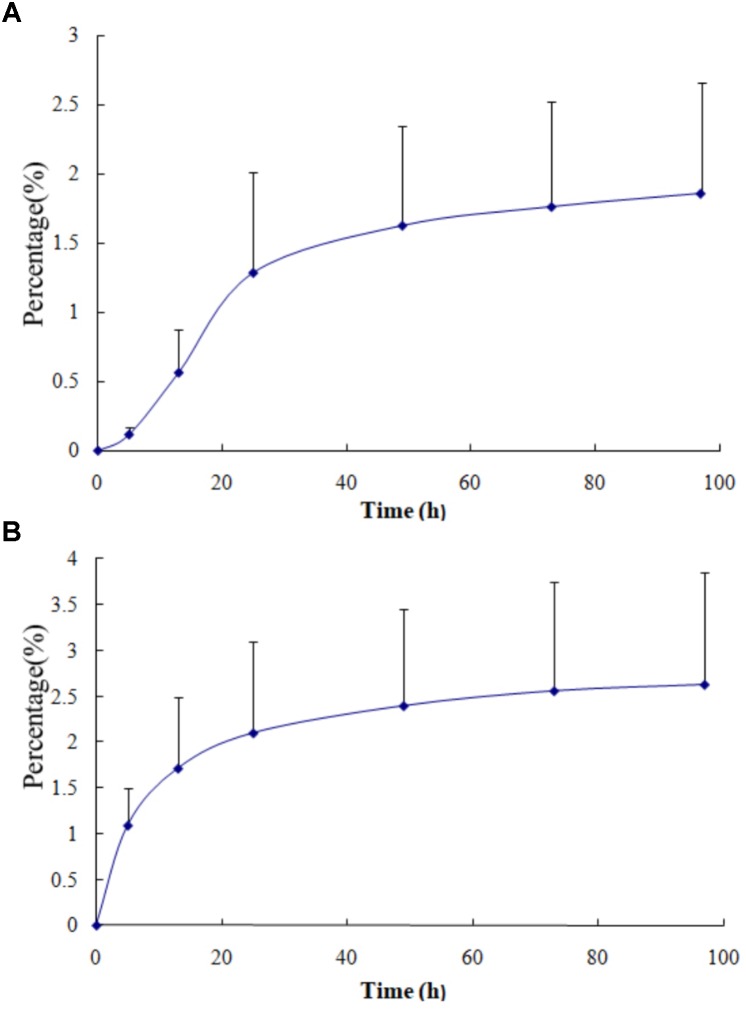
The cumulative urinary excretion of VCR in urine from four patients after dosing with VSLI [1.0 mg/m^2^, **(A)**] and VSI [1.4 mg/m^2^, **(B)**], respectively.

The cumulative excretion percentage of VCR in urine from four patients treated with VSLI (1.0 mg/m^2^) and VSI (1.4 mg/m^2^) are shown in **Figure 3**, respectively. Less than 3% of the injected VSLI dose was eliminated in the urine over the 97-h period in the form of unchanged VCR and the extent of urinary excretion was similar to that after treated with VSI. Notably, though about 70% of the total amount of VCR excreted in the urine was recovered within 25 h after both VSLI and VSI administration, time course of excretion had large difference. The majority of VCR was excreted during the first few hours (40% of total VCR excretion within 0–5 h followed by 21% within 5–13 h and 14% within 13–25 h) post infusion of VSI. In contrast, the VCR excretion rate gradually increased over the 25-h period (6.2, 24, and 39% of the total amount of VCR excreted in the urine within 0–5, 5–13, and 13–25 h period, respectively) after VSLI administration.

### Safety

Adverse events (AEs) were graded according to NCI CTCAE for Adverse Events (version 4.0). AEs mentioned in this paper refer to AEs associated with VALI or VSI (shown in **Table 4**). Eight patients (100%) experienced AEs both in treatment 1and 2, seven (87.5%) and four (50.0%) of whom experienced grade ≥3 AE in treatment 1 and 2, respectively. Hematologic toxicity were the most frequently reported AEs (seven patients, 87.5% and eight patients, 100% in treatment 1 and 2, respectively) followed by metabolic disorder (five patients, 62.5% both in treatment 1and 2) and gastrointestinal reaction (four patients, 50% both in treatment 1and 2). Eight patients showed good tolerance using treatment 1and 2 and there was no significant difference of AEs between two treatments, showing good safety of VSLI at 1.0 mg/m^2^ dose level. No serious AEs occurred in this study.

**Table 4:** Summary of adverse events (AEs) associated with VSLI or VSI reported by frequency.

		Grade	Treatment 1 (*n* = 8)	Treatment 2 (*n* = 8)
Hematologic disorders			7	8
	Oligoleukocythemia		7	8
		4	1	1
		3	4	2
	Neutropenia		6	7
		4	2	1
		3	4	3
	Lymphocytopenia		5	4
		4	0	1
		3	2	2
Metabolic disorder			5	5
	Hyperlipoidemia		3	4
		3	0	1
	Glutamic-oxaloacetic transaminase		1	3
	Glutamic-pyruvic transaminase		1	3
Gastrointestinal disorders			4	4
	Nausea		3	3
	Omitting		1	1
Nervous system disorders			2	1
	Numbness of fingers		1	1
	Headache		1	0

## Discussion

Cross-over design was used in order to minimize the variability between the groups. CHOP or modified CHOP scheme, instead of single-agent VSLI or VSI, was applied in this study considering the patient benefit. A conservative dose of 1.0 mg/m^2^ for VSLI was selected in this study, which is approximately 1.4-fold lower than the recommended dose for traditional VCR for safety reasons, because a new VSLI may lead to unpredictable toxicity owing to different pharmaceutical characteristics, though liposomal VCR seemed to exhibit reduced toxicity in previous clinical study.

The PK characteristic of F-VCR and the release properties of VCR from the liposome in human being were studied. The significant lower C_max_ and AUC_0-Inf_ of F-VCR demonstrated that majority of the VCR was encapsulated in the liposome and the VCR was released slowly from the liposome though the sudden release was observed reflected by multiple peaks of plasma-concentration profiles.

In this study, the higher C_max_ values, longer circulation half-lives, and longer mean residence times observed with the T-VCR of VSLI, compared with VSI, were associated with a significantly higher plasma AUC values for T-VCR. This could be explained that VSLI decreases in plasma clearance rates and the volume of distribution compared with VSI. These data were very similar to those obtained from another study in China (Yan et al., 2012). In addition, the PK parameter values of T-VCR including C_max_, AUC_0-Inf_, CL and V_z_ from the present study were in consistent with those from the study mentioned above (Yan et al., 2012). However, the main PK parameters of T-VCR in this study had large difference from those reported in previous studies from United States (Bedikian et al., 2011) and Canada (Embree et al., 1998; Gelmon et al., 1999). The data, summarized in **Table 5**, showed that the dose-normalized C_max_ and AUC_0-Inf_ value from three international studies were similar and exceeded those from this current study by approximately 5- and 30-fold, respectively. Besides, compared to the current study, the V_z_ of T-VCR in two studies from United States (Bedikian et al., 2011) and Canada (Embree et al., 1998) reduced by 80- and 50-fold, respectively. The CL of T-VCR reduced by 24-fold in study from United States vs. this present study. The differences in pharmaceutical property of VSLI including the ingredient and particle size uniformity of liposome, or in the patients’ characteristics (race, cancer types and disease states) in the three international studies vs. the current study may be responsible for these inconsistencies. In addition, methodological differences used in different studies may contribute to the inconsistencies.

**Table 5:** PK parameters of T-VCR from different studies.

Drug name	Dose level (mg/m^2^)	C_max_	(ng/mL)	AUC_0-Inf_ (ng/mL⋅h)	Vz (L)	CL (L/h)	Patients information				Reference
							Number	Race	Cancer types	Nation	
VSLI	1	86.6 (37.4)	222.1 (86.5)	224.1 (109)	8.9 (4.2)	8	Asian	Malignant lymphoma	China	This present study
VSLI	2	127 (80.4)	242.9 (60.8)	309 (120)	8.7 (2.3)	6	Asian	Advanced solid tumors	China	Yan et al., 2012
Marqibo^®^	1	404 (116)	6407 (2008)	2.8 (1.3)	0.36 (0.1)	7	6 Caucasian and 1 Hispanic	Metastatic melanoma	American	Bedikian et al., 2011
VSLI	2	891 (671)	6483 (613)	4.3 (0.8)	NA	2	NA	Advanced carcinoma	Canada	Embree et al., 1998
ONCO-TCS	1	400 (400)	8300 (8300)	NA	NA	4	NA	Advanced carcinoma	Canada	Gelmon et al., 1999

*NA, not available.*

Two physical states of T-VCR exist in the circulation after VSLI administration including F-VCR (unentrapped and released from liposome) and liposome-entrapped VCR. It is theoretically agreed that F-VCR is responsible for causing toxicity and liposomal VCR accounts for providing the activity. From the safety perspective, it is essential to ensure that the plasma levels of F-VCR post injection of VSLI are comparable with or below those of VCR after treatment with VSI. Thus, it was anticipated that VCR is released from the liposome to increase the concentration of VCR in tumor site and to decrease the concentration of free drug in plasma, then the vast majority of drug present in circulation remains entrapped with liposomes. In order to confirm this hypothesis and better understand the release character of VCR from liposome of VSLI in human being, we developed an SPE method to separate the F-VCR from the liposomal VCR in plasma and the concentration of F-VCR was determined (Yang et al., 2013). Based on these data, we observed that the VCR was not released at constant speed because multiple peaks appeared in the plasma F-VCR concentration-time profile. While, the lower C_max_ showed that the release rate of VCR from lipid carrier was slow and controllable in general, even though the sudden release existed to some degree. When comparing the AUC_0-Inf_ value of F-VCR to that of T-VCR after administration with VSLI, the ratio at 29.2% indicated that there are only a small fraction of VCR released from liposome in the plasma. Furthermore, the lower F-VCR concentration in plasma for VSLI than that of VCR for VSI supplied the theoretical basis for the fact that VSLI lead to decreased toxicity compared to VSI, and this is consistent with the hypothesis.

It is expected that the excretion route of VCR released from liposome was similar to that of traditional VCR. The biliary system is the principal excretion route for traditional VCR (Jackson et al., 1978). To confirm it, we developed a simple method to determine the VCR concentration in urine and calculate the cumulative excretion percentage of VCR in urine (Yang et al., 2015). Less than 3% of the administered dose of VSLI was excreted from urinary post injection and the extent was similar to that for VSI. Nevertheless, both the time course and the elimination rate were very different. The elimination percentage of 40–21–14% for VSI changed to 6.2–24–39% for VSLI at intervals of 0–5, 5–13, and 13–25 h, respectively. Considering the fact that it is impossible for liposomes to be cleared via glomerular filtration due to the size, the elimination rate of VCR from urine should entirely be determined by the release rate of VCR from liposome. Hence, the gradual increasing of excretion rate for VSLI indicated that VCR was slowly released from liposome. In a word, the above data declared that the liposomal VCR did not change the elimination route of VCR, but the elimination rate was different because of the slow release of VCR from liposome.

Vincristine sulfate liposome injection was well tolerated, no serious AEs were reported and no patient was withdrawn due to an AE during the study. The profile and frequency of AE, as well as the numbers of the patients who experienced the grade ≥3 AE for VSLI were consistent with that for conventional VSI. No additional toxicity was obs**erved even** though the 2.8-times higher plasma AUC_0-Inf_ of T-VCR for VSLI than that of VCR for VSI was obtained.

## Conclusion

In summary, the high exposure of T-VCR and low exposure of F-VCR demonstrated that the proportion of F-VCR was very small and most of VCR was entrapped in liposome in circulation following injection of VSLI, which explained the fact that VSLI showed higher efficacy and lower toxicity than that for VSI. VCR was released from liposome slowly, which was reflected by the low plasma concentration of F-VCR and the delayed excretion of VCR in urine. Liposomal encapsulation of VCR did not alter the extent of VCR excretion in urine, but time course changed.

## Ethics Statement

The phase Ia period (Clinical Trials.gov Identifier: 2009L01014) was conducted at three sites in China. It was approved by the Ethics Committee of Peking Union Medical College Hospital (Site 1, Permission Number: 1254), Nanjing First Hospital Affiliated of Nanjing Medical University (Site 2, Permission Number: 2011-MD- I22) and The Second Affiliated Hospital of Soochow University (Site 3, Permission Number: 2012-3), respectively. The study was conducted in accordance with the principles of the Declaration of Helsinki and Good Clinical Practice guidelines. The written Informed Consent Form was obtained from each subject prior to any study procedure.

## Author Contributions

FY and MJ contributed equally to the study design, process of samples, data analysis, and article writing. ML assisted with the process of samples and data acquisition. PH was involved in the design of the study and article writing. JJ and HW were the principal investigators of this trial and were involved in the study design and analysis of data. All authors revised the article critically and gave final approval of the version to be published.

## Conflict of Interest Statement

The authors declare that the research was conducted in the absence of any commercial or financial relationships that could be construed as a potential conflict of interest.

## Supplementary Material

FIGURE S1 F-VCR concentration-time curve for subject 1.

FIGURE S2 F-VCR concentration-time curve for subject 2.

FIGURE S3 F-VCR concentration-time curve for subject 3.

FIGURE S4 F-VCR concentration-time curve for subject 4.

FIGURE S5 F-VCR concentration-time curve for subject 5.

FIGURE S6 F-VCR concentration-time curve for subject 6.

FIGURE S7 F-VCR concentration-time curve for subject 7.

FIGURE S8 F-VCR concentration-time curve for subject 8.

## Abbreviations

AE: adverse event
AUC_0-Inf_: area under plasma concentration-time curve from zero to infinity
C_max_: maximum concentration
CHOP: cytoxan (cyclophosphamide power injection), hydroxyrubicin (adriamycin power injection), oncovin (vincristine sulfate injection) and prednisone tablets
CL: clearance
CTCAE: Common Terminology Criteria for Adverse Events
F-VCR: free vincristine
IS: internal standard
I.V.: intravenously
NCI: National Cancer Institute
PK: pharmacokinetic
SPE: solid phase extraction
T-VCR: total vincristine
UPLC-MS/MS: ultra-performance liquid chromatography-tandem mass spectrometry
VSI: vincristine sulfate injection
VSLI: vincristine sulfate liposomes injection
V_z_: distribution volume
t_1/2_: elimination half-life
