# Supplementary figures and images for: Pharmacokinetic Behavior of Vincristine and Safety Following Intravenous Administration of Vincristine Sulfate Liposome Injection in Chinese Patients With Malignant Lymphoma
Source: Front Pharmacol. 2018 Aug 29;9:991. doi: 10.3389/fphar.2018.00991 (PMC6123375; doi:10.3389/fphar.2018.00991)

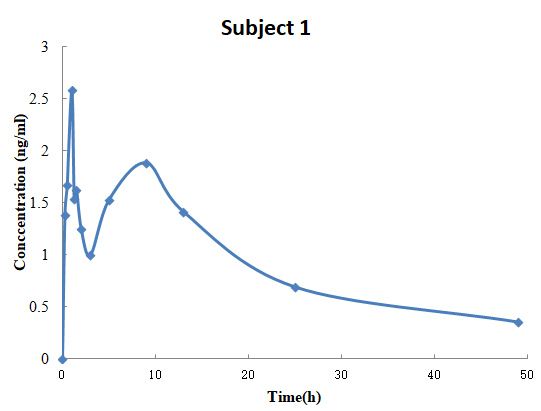

Supplement: FIGURE S1 — F-VCR concentration-time curve for subject 1. [file Image_1.JPEG]

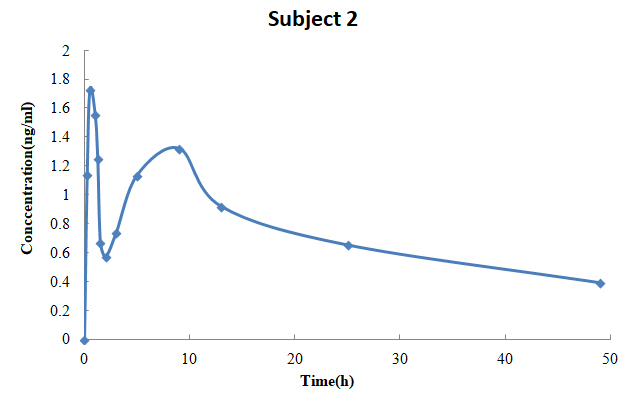

Supplement: FIGURE S2 — F-VCR concentration-time curve for subject 2. [file Image_2.JPEG]

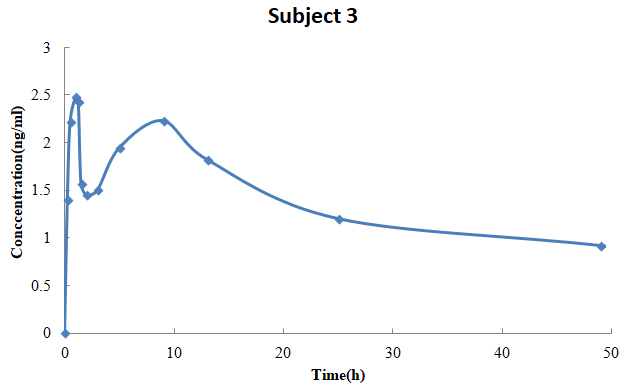

Supplement: FIGURE S3 — F-VCR concentration-time curve for subject 3. [file Image_3.JPEG]

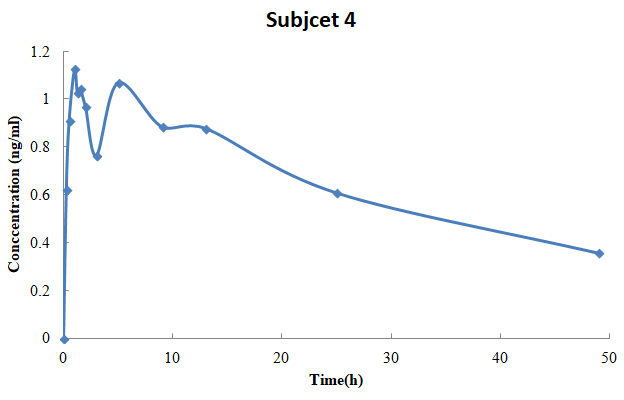

Supplement: FIGURE S4 — F-VCR concentration-time curve for subject 4. [file Image_4.JPEG]

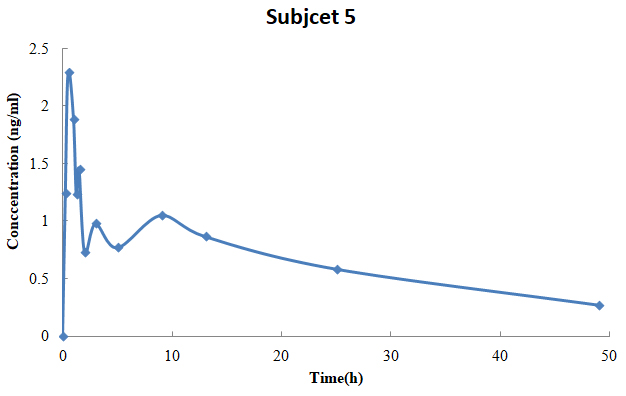

Supplement: FIGURE S5 — F-VCR concentration-time curve for subject 5. [file Image_5.JPEG]

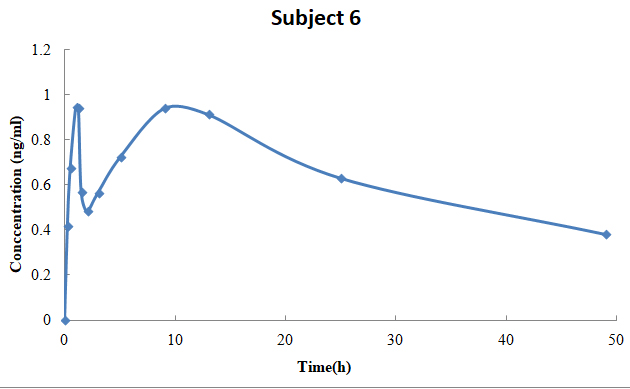

Supplement: FIGURE S6 — F-VCR concentration-time curve for subject 6. [file Image_6.JPEG]

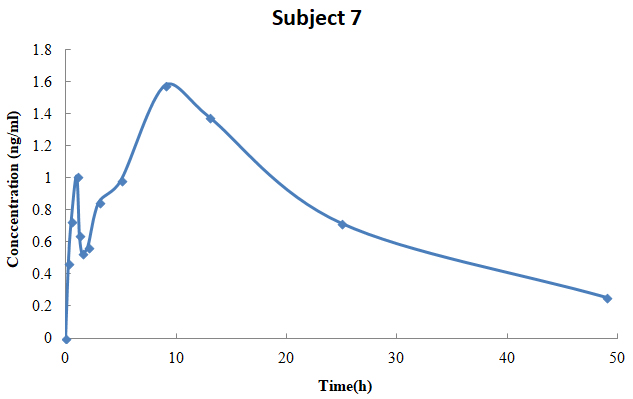

Supplement: FIGURE S7 — F-VCR concentration-time curve for subject 7. [file Image_7.JPEG]

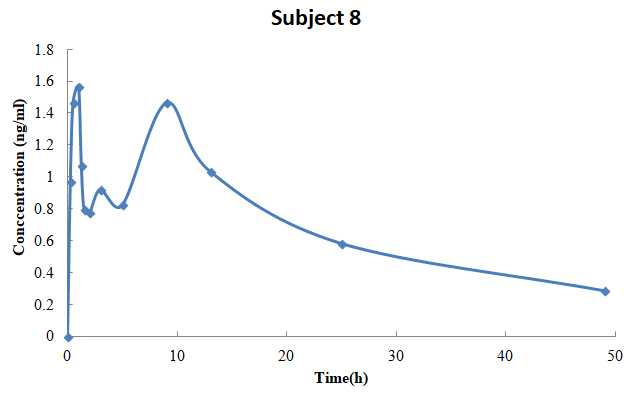

Supplement: FIGURE S8 — F-VCR concentration-time curve for subject 8. [file Image_8.JPEG]
